# Comprehensive *FGFR3* alteration-related transcriptomic characterization is involved in immune infiltration and correlated with prognosis and immunotherapy response of bladder cancer

**DOI:** 10.3389/fimmu.2022.931906

**Published:** 2022-07-26

**Authors:** Ting Xu, Weizhang Xu, Yuxiao Zheng, Xiao Li, Hongzhou Cai, Zicheng Xu, Qing Zou, Bin Yu

**Affiliations:** Department of Urology, Jiangsu Cancer Hospital & Jiangsu Institute of Cancer Research & Affiliated Cancer Hospital of Nanjing Medical University, Nanjing, China

**Keywords:** bladder cancer, fibroblast growth factor receptor 3, *FGFR3* alteration-related genes (FARGs), overall survival, nomogram, immune infiltration, immunotherapy response, chemotherapy response

## Abstract

**Background:**

Bladder cancer (BC) threatens the health of human beings worldwide because of its high recurrence rate and mortality. As an actionable biomarker, fibroblast growth factor receptor 3 (*FGFR3*) alterations have been revealed as a vital biomarker and associated with favorable outcomes in BC. However, the comprehensive relationship between the *FGFR3* alteration associated gene expression profile and the prognosis of BC remains ambiguous.

**Materials and Methods:**

Genomic alteration profile, gene expression data, and related clinical information of BC patients were downloaded from The Cancer Genomics database (TCGA), as a training cohort. Subsequently, the Weighted Gene Co-expression Network Analysis (WGCNA) was conducted to identify the hub modules correlated with *FGFR3* alteration. The univariate, multivariate, and least absolute shrinkage and selection operator (LASSO) Cox regression analyses were used to obtain an *FGFR3* alteration-related gene (FARG) prognostic signature and FARG-based nomogram. The receiver operating characteristic (ROC) curve analysis was used for evaluation of the ability of prognosis prediction. The FARG signature was validated in four independent datasets, namely, GSE13507, GSE31684, GSE32548, and GSE48075, from Gene Expression Omnibus (GEO). Then, clinical feature association analysis, functional enrichment, genomic alteration enrichment, and tumor environment analysis were conducted to reveal differential clinical and molecular characterizations in different risk groups. Lastly, the treatment response was evaluated in the immunotherapy-related dataset of the IMvigor210 cohort and the frontline chemotherapy dataset of GSE48276, and the chemo-drug sensitivity was estimated *via* Genomics of Drug Sensitivity in Cancer (GDSC).

**Results:**

There were a total of eleven genes (*CERCAM*, *TPST1*, *OSBPL10*, *EMP1*, *CYTH3*, *NCRNA00201*, *PCDH10*, *GAP43*, *COLQ*, *DGKB*, and *SETBP1*) identified in the FARG signature, which divided BC patients from the TCGA cohort into high- and low-risk groups. The Kaplan–Meier curve analysis demonstrated that BC patients in the low-risk group have superior overall survival (OS) than those in the high-risk group (median OS: 27.06 months *vs*. 104.65 months, *p* < 0.0001). Moreover, the FARG signature not only showed a good performance in prognosis prediction, but also could distinguish patients with different neoplasm disease stages, notably whether patients presented with muscle invasive phenotype. Compared to clinicopathological features, the FARG signature was found to be the only independent prognostic factor, and subsequently, a FARG-based prognostic nomogram was constructed with better ability of prognosis prediction, indicated by area under ROC curve (AUC) values for 1-, 3-, and 5-year OS of 0.69, 0.71, and 0.79, respectively. Underlying the FARG signature, multiple kinds of metabolism- and immune-related signaling pathways were enriched. Genomic alteration enrichment further identified that *FGFR3* alterations, especially c.746C>G (p.Ser249Cys), were more prevalent in the low-risk group. Additionally, FARG score was positively correlated with ESTIMATE and TIDE scores, and the low-risk group had abundant enrichment of plasma B cells, CD8+ T cells, CD4+ naive T cells, and helper follicular T cells, implying that patients in the low-risk group were likely to make significant responses to immunotherapy, which was further supported by the analysis in the IMvigor210 cohort as there was a significantly higher response rate among patients with lower FARG scores. The analysis of the GDSC database finally demonstrated that low-risk samples were more sensitive to methotrexate and tipifarnib, whereas those in the high-risk group had higher sensitivities in cisplatin, docetaxel, and paclitaxel, instead.

**Conclusion:**

The novel established FARG signature based on a comprehensive *FGFR3* alteration-related transcriptomic profile performed well in prognosis prediction and was also correlated with immunotherapy and chemotherapy treatment responses, which had great potential in future clinical applications.

## Introduction

Bladder cancer (BC) is one of the most common malignant cancers worldwide (1, 2), with increasing newly diagnosed and death cases by year (3–5), which is also the main cause of genitourinary cancer-related deaths. BC generally originates from the epithelium, mainly classified into two subtypes, namely, muscle-invasive bladder cancer (MIBC) and non-muscle-invasive bladder cancer (NMIBC), according to the extent of the tumor penetrating the bladder wall. As is reported, approximately 75% of the BC patients are diagnosed with NMIBC while 25% are generally MIBC, with each having distinct molecular drivers (6). The conventional treatment options, including surgery, radiation, and cisplatin-based chemotherapy, have been widely used, but there still exist high rates of recurrence and metastasis within BC patients (7). Currently, the genomic, transcriptomic, and proteomic sequencing efforts provide in-depth insights into BC biology, having made great progresses in characterizing BC patients into different molecular subtypes and promoting the targeted treatments and immunotherapies. However, these advances are limited to small-scale BC patients, and the comprehensive molecular characterization remained to be fully elucidated, especially in identifying a novel prognostic biomarker improving clinical management of BC and an effective predictor for treatment response.

The aberrant FGFR signaling axis, including extracellular signal-regulated kinase (ERK)/mitogen-activated protein kinase (MAPK) pathways, is usually considered to be associated with oncogenesis and tumor progression in different cancer types (8). Over the past decades, fibroblast growth factor receptor (*FGFR*) alterations have been widely identified in BC, breast cancer, glioblastoma, intrahepatic cholangiocarcinoma, lung cancer, as well as many other malignancies (9). As a member of the structurally related tyrosine kinase receptors family, *FGFR3* is highly conserved among divergent species, and located on chromosome 4p16.3, consisting of 19 exons and 18 introns (10). Interestingly, *FGFR3* alterations are mainly prevalent in BC, which has been identified as one of the most frequently altered genes in nearly 40% of BC patients (11, 12). Simultaneously, BC patients with *FGFR3* alterations are remarkably correlated with lower tumor stages and grades, are genetically stable, and have longer disease-specific survival (13–15). Additionally, it has been demonstrated that BC patients with *FGFR3* alterations are more sensitive to the treatment of the pan-FGFR inhibitor, erdafitinib (16); under a consensus molecular classification, these kinds of patients are considered to be highly enriched in the luminal subtype, showing poorer response to immune checkpoint inhibitors (ICIs) (17, 18). Controversially, a recent study reveals that there is no difference in survival between ICI-treated patients harboring *FGFR3* alterations or not; neither was there a difference in the complete/partial response (CR/PR) rate (19). Overall, how altered *FGFR3* functions to affect tumor biological behaviors and survival of BC patients is still unclarified, and whether *FGFR3* alterations related to molecular mechanisms correlated with the prognosis and treatment response in BC merits further investigation.

In this study, a novel prognostic FARG, which could robustly divide BC patients into high- and low-risk groups, was established. The underlying FARG signature, the identification of molecular features, and the evaluation of treatment response could further help promote clinical management and provide some potential treatment strategies for BC patients. Moreover, the comprehensive *FGFR3* alteration-related transcriptomic profile was highly correlated with survival of BC patients and affect their immune infiltrations; furthermore, its predictive role in immunotherapy response and chemotherapy response was also deeply investigated.

## Materials and methods

### Data sources and acquisition

The genomic alternation profile, mRNA expression data, and corresponding clinical feature information [including diagnosis age, gender, survival time, neoplasm disease stage (I, II, III, and IV), tumor stage (T stage), lymph node stage (N stage), and metastasis stage (M stage)] of BC patients were downloaded from The Cancer Genomics Atlas (TCGA-BC, Firehose Legacy) *via* the cBioPortal (https://www.cbioportal.org/). The TCGA-BC cohort was chosen as the training group, while GSE13507, GSE31684, GSE32548, and GSE48075 datasets from the Gene Expression Omnibus (GEO) database (http://www.ncbi.nlm.nih.gov/geo/) served as the validation groups.

### Identification of hub modules by weighted gene co-expression network analysis

The DEGs between *FGFR3*-altered and wild-type samples from the TCGA-BC cohort were initially identified, with the fold change > 1.5 and an adjusted *p*-value < 0.01 set as the threshold value. The volcano plot and hierarchical clustering were performed by using the “ggpubr” and “pheatmap” packages in R studio, respectively.

The weighted gene co-expression network analysis (WGCNA), an analysis method, can convert expression data into co-expression gene modules and disclose the relationship between the separated modules and phenotypic traits (20). In the present study, the co-expression network of the DEGs was constructed with the soft thresholding power value of 10, which was decided by a scale free *R* ^2^ of 0.85. Then, the DEGs were classified into different gene modules according to the topological overlap matrix (TOM)-based dissimilarities. The significant modules that were highly correlated with *FGFR3* alterations (*p* < 0.05), were selected for survival analysis, eventually identifying the hub modules associated with OS of BC patients.

### Establishment and validation of FARG signature

The genes involved in the hub modules were then enrolled in the univariate Cox regression analysis, and OS-related hub genes were further employed for the LASSO analysis, which is a form of penalized regression on screening variables from high-dimensional genes to construct risk models (21). The OS-related hub genes were filtered to find the most fit genes with prognostic power by LASSO analysis, finally identifying 11 genes involved in the FARG signature. The optimal value of the tuning parameter (λ) was determined by 10-fold cross-validation using minimum criteria. The FARG score was then calculated using the following formula:

FARG score = sum (expression level of each gene × corresponding coefficient)

According to the median FARG score, BC patients from the TCGA cohort were classified into high- and low-risk groups. The survival analysis and the receiver operating characteristic (ROC) curve analysis were conducted to testify the performance of FARG signature in prognosis prediction. The additional independent datasets of GSE13507, GSE31684, GSE32548, and GSE48075 were retrieved as the validation groups. Subsequently, we retrieved MIBC patients from these four validation groups for further verification of FARG signature, and remarkably, the mRNA expression data were normalized according to the expression of tubulin as internal reference. Additionally, the correlation analysis was conducted to figure out the differentiation, between high- and low-risk groups, of clinical features including gender, diagnosis age, neoplasm disease stage, and T, N, and M stage.

### Construction of the FARG signature-based nomogram

The multivariate Cox regression analyses were performed to determine the prognostic merits of the FARG signature and four clinical features significantly correlated with the FARG score; as a result, the FARG signature was found to be an independent prognostic factor. Subsequently, the FARG signature with combination of neoplasm disease stage, T stage, N stage, and M stage was selected to construct a prognostic nomogram by using the “rms” package in R studio. Meanwhile, calibration curves and time-dependent ROC curves were applied to assess the consistency between predicted and actual survival outcomes.

### Biological function enrichment analysis

The Kyoto Encyclopedia of Genes and Genomes (KEGG) and the Gene ontology (GO, including a total of three categories: BP: biological process, CC: cellular component, MF: molecular function) pathway enrichment analyses were performed to explore the differentiation of biological processes between high- and low-risk groups. The gene set enrichment analysis was conducted by using the “fgsea” package to calculate the enrichment scores, with adjusted *p*-values < 0.05 considered as statistically significant.

### Genomic alterations and TME associated with FARG signature

Genomic alteration profiles were used to analyze and visualize the differences in genomic alterations between high- and low-risk groups by the “maftools” package in R studio. Meanwhile, the alteration frequency of *FGFR3* between high- and low-risk groups was displayed in the lollipop plot. Moreover, stromal score, immune score, and ESTIMATE score were calculated by the ESTIMATE algorithm (22); furthermore, the T-cell dysfunction and exclusion (TIDE) score was used to indicate tumor immune evasion, which can predict the clinical response in treatment of potential immune checkpoint blockade (23). Additionally, 22 common tumor-infiltrated lymphocytes (TILs) were assessed by the CIBERSORT algorithm (24). The Cancer Immunome Atlas (TCIA, https://tcia.at/home) was employed to evaluate the tumor purity of different risk groups (25, 26).

### Evaluation of immunotherapy response and chemotherapy drug sensitivity analysis

In the present study, the IMvigor210 cohort, a group of BC patients treated by immunotherapy with anti-PD-L1 therapy of atezolizumab, was further employed to predict the treatment response to programmed death-ligand 1 (PD-L1, also known as CD274) blockade. The treatment response was defined as the following criteria: CR: complete response, PR: partial response, SD: stable disease, and PD: progressive disease.

The GSE48276 dataset, including luminal and basal subtypes of MIBC patients treated with frontline chemotherapy (MVAC treatment: Methotrexate, Vinblastine, Adriamycin, Cisplatin), was retrieved to evaluate the treatment response of frontline chemotherapy. The database of Genomics of Drug Sensitivity in Cancer (GDSC, https://www.cancerrxgene.org/) was utilized to predict the sensitivity of chemotherapy treatment by using the package “pRRophetic” in R studio (27). The IC_50_ (half-maximal inhibitory concentration) values of chemo-drugs, including bleomycin, cisplatin, docetaxel, doxorubicin, gemcitabine, methotrexate, paclitaxel, tipifarnib, vinblastine, vinorelbine, and vorinostat, were collected to investigate the correlation between FARG score and chemotherapy drug sensitivity. The above chemo-drug sensitivity analysis was conducted in human cancer cell lines, but which could predict the response of chemotherapeutic treatment between different risk groups to some extent.

### Statistical analysis

All statistical analyses in this study were conducted in R studio (https://rstudio.com/). The Kruskal–Wallis, Mann–Whitney *U*, and Fisher’s exact tests were devoted to comparing the categorical variables, and survival analysis by using log rank test was conducted to evaluate the differences in survival rates between different risk groups. The results of multivariate Cox regression analysis were visualized with a nomogram. Concordance index (C-index), time-dependent ROC curve, calibration curve, and decision curve (DC) analyses were also important indicators used to assess the nomogram. For all statistical analyses, (adjust) *p*-value < 0.05 was considered statistically significant.

## Results

### Patient sample characteristics

In this study, a total of 282 MIBC patient samples, having genomic alteration profiles and mRNA expression data, were selected from the TCGA-BC cohort. Four independent datasets, including GSE13507 (165 BC samples with primary tumors were included), GSE31684 (93 BC samples), GSE32548 (130 BC samples), and GSE48075 (73 BC samples), were used as the validation groups; of note, patient samples without survival time were excluded. The detailed information of clinical features of BC patients from TCGA-BC and GEO datasets is shown in **Table 1**.

**Table 1:** Clinical characteristics of the BC patients in this study.

Characteristics	TCGA-BC	GSE13507	GSE31684	GSE32548	GSE48075
**Used samples (Total)**	282 (476)	165 (256)	93 (93)	130 (131)	73 (142)
Age (median)	69	66	69	71	69
**Gender**					
Male	203	135	68	100	54
Female	79	30	25	31	19
**Histological type**					
MIBC	282	62	78	38	73
NMIBC	0	103	15	93	0
**Neoplasm disease stage**					
Stage I	0	NA	NA	NA	NA
Stage II	91	NA	NA	NA	NA
Stage III	97	NA	NA	NA	NA
Stage IV	93	NA	NA	NA	NA
**T stage**					
T0/Ta/Tis	1	24	5	41	1
T1	1	80	10	52	0
T2	83	31	17	38	41
T3	131	19	42	0	23
T4	43	11	19	0	8
**N stage**					
N0	158	149	49	NA	62
N1	29	8		NA	11
N2	56	6	28	NA	
N3	5	1		NA	
**M stage**					
M0	135	158	57	NA	66
M1	8	7	36	NA	7

A total of 282 BC patients with both genomic alteration information and mRNA expression data from the TCGA cohort were selected. In GSE13507, 165 BC samples with primary tumors were included. In GSE32548 and GSE48075, BC patients without survival information were excluded. MIBC, muscle-invasive bladder cancer; NMIBC, non- muscle-invasive bladder cancer; NA, not applicable.

### Identification of the key modules and hub genes

**Figure 1:**
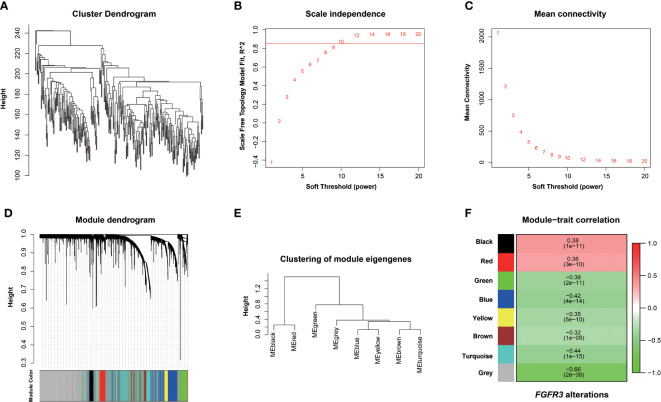
Screening *FGFR3* alterations related to modules by the Weighted Gene Co-expression Network Analysis (WGCNA). **(A)** Hierarchical clustering based on mRNA expressions of 282 BC patients from TCGA. **(B)** Determination of power value in the WGCNA analysis. **(C)** The association between power value and connection degree. **(D)** Clustering and merging the co-expression modules. **(E)** Clustering of module eigengenes. **(F)** The heatmap of co-expression module-*FGFR3* alteration correlation; red and green represent positive and negative correlation, respectively.

**Figure 2:**
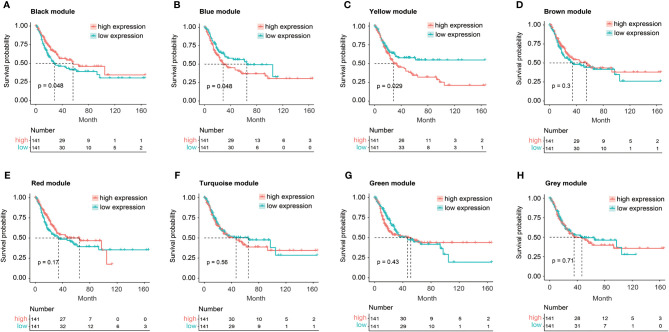
The Kaplan–Meier curve analysis for BC patients with distinct expression pattern when exploring the relationship between eight key modules and overall survival (OS) of BC patients from TCGA. **(A)** The expression of black module was positively correlated with OS of BC patients. **(B, C)** The expression of blue **(B)** or yellow **(C)** module was negatively correlated with OS of BC patients. (D–H) No statistically significant difference in OS of BC patients between high and low expression of brown **(D)**, red **(E)**, turquoise **(F)**, green **(G)**, or gray **(H)** module.

The relationship between *FGFR3* status and OS of BC patients was initially investigated, however, no significant difference in the OS was observed between *FGFR3*-altered and wild-type groups (*p* = 0.29, **Supplementary Figure 1**). As *FGFR3* alteration was still a widely acknowledged favorable prognostic factor, a total of 3,724 DEGs were subsequently identified between 42 *FGFR3*-altered and 240 wild-type BC patient samples from the TCGA-BC cohort, of which 962 were upregulated and 2,762 were downregulated (fold change > 1.5, adjusted *p*-value < 0.01, **Supplementary Figure 2**). All the DEGs were subsequently enrolled in the WGCNA with a soft threshold power value of 10 (**Figures 1A–E**). Of note, a total of eight key modules, including a gray module composed of the unclassified genes, were identified to be significantly correlated with *FGFR3* alterations (red and green color represented positive and negative correlation, respectively, *p* < 0.0001, **Figure 1F**). The survival analysis by Kaplan–Meier curve (**Figures 2A–H**) showed that higher expression levels of genes involved in the black module were correlated with prolonged OS of BC patients (median OS: 56.48 months *vs*. 28.24 months, *p* = 0.048, **Figure 2A**); on the contrary, higher expression levels of genes involved in the blue and yellow modules were markedly associated with worse OS (median OS in blue module: 28.41 months *vs*. 64.80 months, *p* = 0.048, **Figure 2B**; in yellow module: median OS: 28.40 months *vs*. not reached, *p* = 0.029, **Figure 2C**). Collectively, a total of 966 genes involved in these three modules were selected as the hub genes correlated with OS of BC patients (**Supplementary Table 1**).

### Establishment and validation of a novel FARG signature

**Figure 3:**
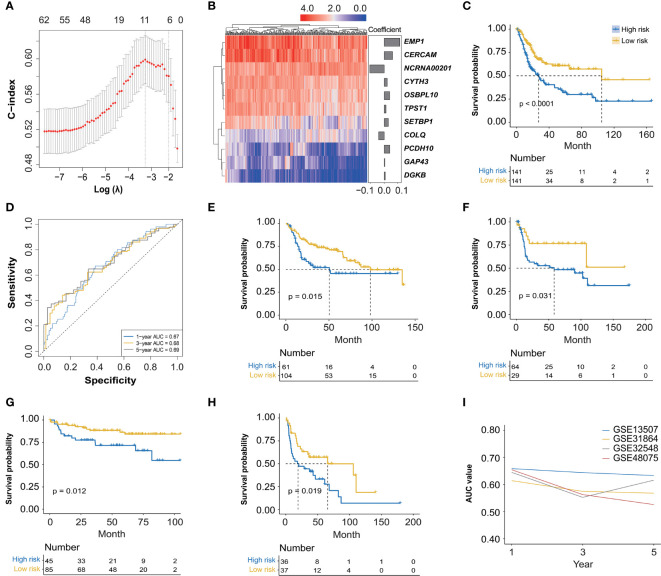
The construction and validation of a novel *FGFR3* alteration-related gene (FARG) signature. **(A)** A total of 11 FARGs were involved in the prognostic signature by the least absolute shrinkage and selection operator (LASSO) Cox regression analysis. **(B)** The heatmap of these 11 FARGs’ expression among BC patients and their coefficients were exhibited. **(C)** The Kaplan–Meier curve analysis for high- and low-risk BC patients, which were divided by the median value of the FARG score. **(D)** The receiver operating characteristic (ROC) curve analysis for evaluation of the novel constructed FARG signature. **(E–H)** The FARG signature was further validated in four independent cohorts: GSE13507 **(E)**, GSE31684 **(F)**, GSE32548 **(G)**, and GSE48075 **(H)**. **(I)** The area under ROC curve (AUC) values at 1-, 3-, and 5-year OS further verified the good performance of the FARG signature in prognosis prediction.

The association analysis between these identified 966 hub genes and OS of BC patients was then conducted by the univariate Cox regression analysis, finally identifying 62 OS-related hub genes (**Supplementary Table 2**, *p* < 0.01), which were further enrolled in the LASSO Cox regression analysis (**Figure 3A**), and there were 11 genes selected (*CERCAM*, *TPST1*, *OSBPL10*, *EMP1*, *CYTH3*, *NCRNA00201*, *PCDH10*, *GAP43*, *COLQ*, *DGKB*, and *SETBP1*) to establish a novel FARG signature (**Figure 3B**). The FARG score for each BC patient was calculated according to the following formula:

FARG score = 0.062 × expression level of *CERCAM* + 0.013 × expression level of *TPST1* + 0.041 × expression level of *OSBPL10* + 0.116 × expression level of *EMP1* + 0.023 × expression level of *CYTH3* − 0.107 × expression level of *NCRNA00201* + 0.042 × expression level of *PCDH10* + 0.004 × expression level of *GAP43* − 0.041 × expression level of *COLQ* + 0.008 × expression level of *DGKB* + 0.030 × expression level of *SETBP1*

According to the median FARG score, BC patients from the TCGA cohort were divided into high-and low-risk groups, and Kaplan–Meier curve analysis demonstrated that BC patients in the low-risk group had a longer OS than those in the high-risk group (median OS: 27.06 months *vs*. 104.65 months, *p* < 0.0001, **Figure 3C**). Meanwhile, the time-dependent ROC curve analysis showed that the AUC values of the FARG signature at 1-, 3-, and 5-year OS were 0.67, 0.68, and 0.69, respectively (**Figure 3D**). Subsequently, this novel established FARG signature was verified in four independent validation groups (GSE13507, GSE31684, GSE32548, and GSE48075), retrieved from the GEO database. The Kaplan–Meier curve analysis demonstrated that the FARG signature could successfully distinguish patients with different OS in all four validation groups (GSE13507: median OS: 50.4 months *vs*. 98.0 months, *p* = 0.015, **Figure 3E**; GSE31684: median OS: 58.3 months *vs*. not reached, *p* = 0.031, **Figure 3F**; GSE32548: median OS: not reached *vs*. not reached, *p* = 0.012, **Figure 3G**; GSE48075: median OS: 19.3 *vs*. 65.6 months, *p* = 0.019, **Figure 3H**). Additionally, the AUC values at 1-, 3-, and 5-year OS in these four independent validation groups further revealed that the FARG signature was reliable and robust in the prognosis prediction (**Figure 3I**).

### Prognostic values of FARG signature-related genes in BC

**Figure 4:**
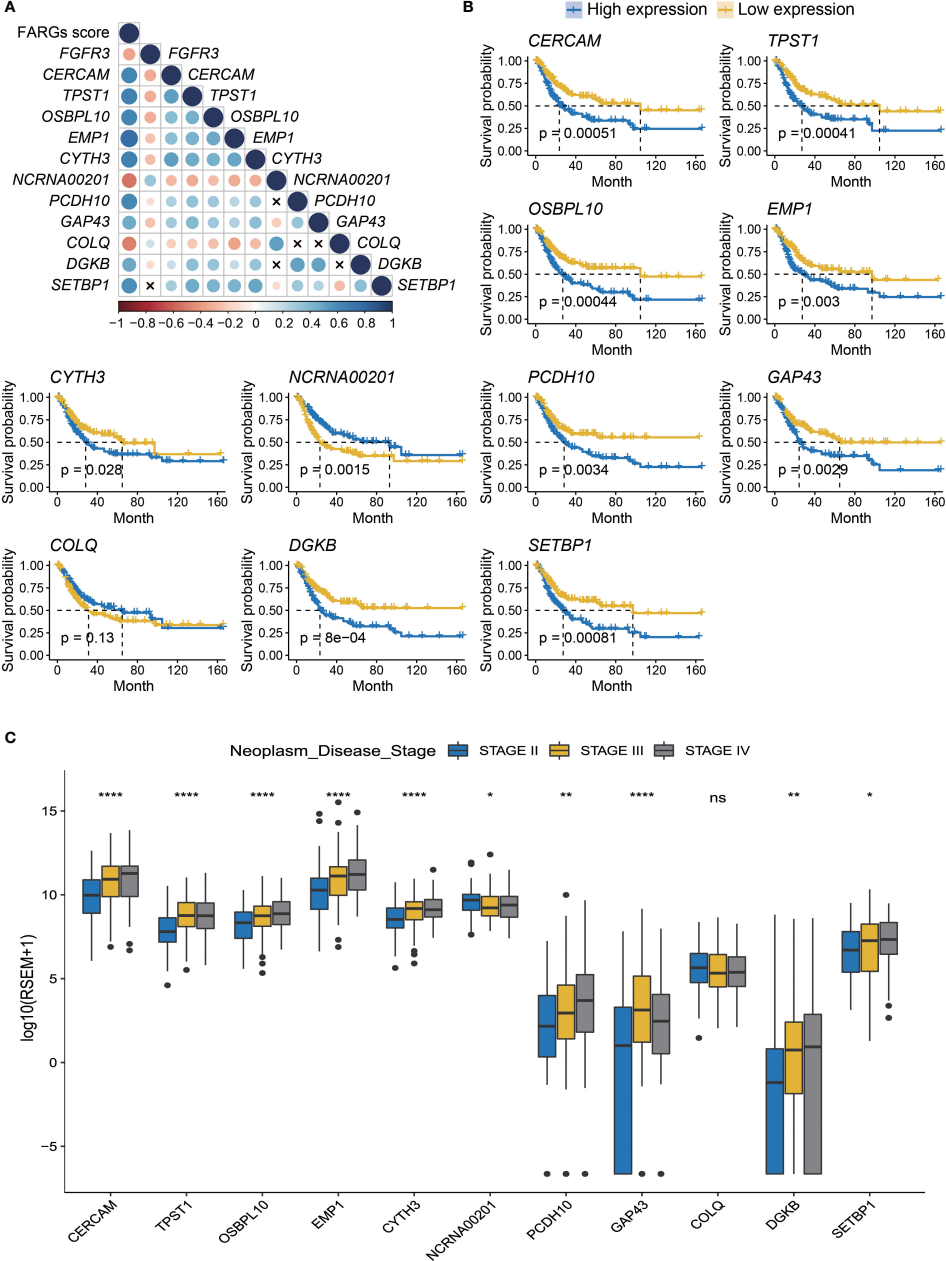
The prognostic merits of 11 FARGs involved in the signature. **(A)** The correlation analysis of *FGFR3* expression and 11 signature-related FARGs (*CERCAM*, *TPST1*, *OSBPL10*, *EMP1*, *CYTH3*, *NCRNA00201*, *PCDH10*, *GAP43*, *COLQ*, *DGKB*, and *SETBP1*) at the transcriptional level (× indicated correlations were not significant). **(B)** The Kaplan–Meier curve analysis for BC patients with high and low expression of these 11 signature-related FARGs. **(C)** The comparison of expression of 11 signature-related FARGs between BC patients in different neoplasm disease stages. * indicated *p* < 0.05, ** indicated *p* < 0.01, **** indicated *p* < 0.0001, ns indicated no statistically significant difference.

Additionally, we analyzed the expression level of these eleven genes involved in FARG signature and found that nine of them were significantly increased in the high-risk group, whereas *NCRNA00201* and *COLQ* presented higher expression level in the low-risk group (*p* < 0.0001, **Supplementary Figure 3**). Moreover, correlation analysis revealed that *CERCAM*, *TPST1*, *OSBPL10*, *EMP1*, *CYTH3*, *PCDH10*, *GAP43*, *DGKB*, and *SETBP1* had positive correlation with each other but negative correlation with *FGFR3*, *NCRNA00201*, and *COLQ* at the transcriptional level (**Figure 4A**). The Kaplan–Meier curve analysis showed that low expression of *CERCAM*, *TPST1*, *OSBPL10*, *EMP1*, *CYTH3*, *PCDH10*, *GAP43*, *DGKB*, and *SETBP1* was highly correlated with the longer OS of BC patients (**Figure 4B**). On the contrary, the decreased expression of *NCRNA00201* was significantly correlated with the shorter OS (*p* < 0.01), while statistical analysis showed no correlation between the expression level of *COLQ* and OS of BC patients (*p* = 0.13, **Figure 4B**). In addition, it was found that there was a statistically significant difference in the expression of nearly all FARG signature-related genes between different neoplasm disease stages, except *COLQ* (*p* < 0.05, **Figure 4C**). Meanwhile, most of these genes were significantly overexpressed in the tumor with advanced neoplasm disease stages, except *NCRNA00201*.

### Association between FARG signature and clinical features

**Figure 5:**
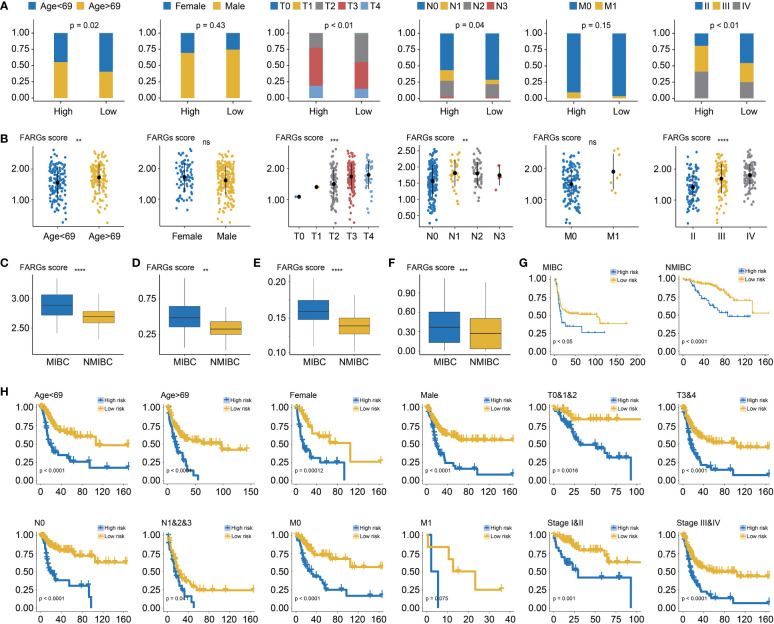
The correlation analysis between the FARG signature and clinical features. **(A)** The clinical association analysis demonstrated that more advanced BC patients were enriched in the high-risk group. **(B)** The FARG signature score was positively correlated with advanced clinical status. **(C)** Muscle-invasive BC (MIBC) patients had higher FARG scores than NMIBC patients in GSE13507. **(D)** MIBC patients had higher FARG scores than NMIBC patients in GSE31684. **(E)** MIBC patients had higher FARG scores than NMIBC patients in GSE32548. **(F)** The comparison analysis of FARG score between MIBC and NMIBC groups by integrating MIBC or NMBIC patients from GSE13507, GSE31684, and GSE32548. **(G)** Kaplan–Meier curve analysis, in the cohort of merging GSE13507, GSE31684, and GSE32548 datasets, for high- and low-risk groups among MIBC patients or NMIBC patients. **(H)** The stratification analysis revealed the robust ability of the FARG signature in prognosis prediction, which also exhibited the potential to predict OS for early-stage BC patients. ** indicated *p* < 0.01, *** indicated *p* < 0.001, **** indicated *p* < 0.0001, ns indicated no statistically significant difference.

Among BC patients from the TCGA-BC cohort, the clinical feature analysis (**Figure 5A**) revealed that significantly more BC patients in the high-risk group were diagnosed at age over 69 years old than the low-risk group (55.32% *vs*. 40.43%, *p* = 0.02). Underlying the FARG signature, it was observed that the gender proportion between two groups was nearly equivalent (*p* = 0.43). Furthermore, it was found that there was a bigger number of patients in the T3 and T4 stage in the high-risk group (high *vs* low: 77.54% *vs*. 55.37%, *p* < 0.01), as well as more patients with lymph node metastasis (43.41% *vs*. 29.57%, *p* = 0.04). However, there was no significant difference in patient number of distant metastasis between two groups (high *vs* low: 9.26% *vs*. 3.37%, *p* = 0.15), probably due to the limited number of patients with phenotypic distant metastasis. The investigation of association between FARG signature and neoplasm disease stage also further revealed that more patients with advanced stages were included in the high-risk group (stage I: none *vs*. none, stage II: 19.15% *vs*. 45.71%, stage III: 39.72% *vs*. 29.29%, stage IV: 41.13% *vs*. 25.00%, *p* < 0.01). Correspondingly, diagnosis age, T stage, N stage, and neoplasm disease stage were positively correlated with the FARG score (*p* < 0.05, **Figure 5B**).

Of note, it was found that MIBC samples had higher FARG scores than NMIBC samples in the GSE13507 (*p* < 0.0001, **Figure 5C**), GSE31684 (*p* < 0.01, **Figure 5D**), and GSE32548 cohorts (*p* < 0.0001, **Figure 5E**), implying that FARG signature could also predict the phenotypic muscle-invasiveness status. After merging MIBC and NMIBC patients from the GSE13507, GSE31684, and GSE32548, it was also found that MIBC samples had significantly higher FARG scores (*p* < 0.001, **Figure 5F**). Of note, the FARG signature not only exhibited good performance in prognosis prediction for MIBC patients (median OS: 15.47 months *vs*. 102.70 months, *p* < 0.05) but also predicted prognosis for NMIBC patients well (median OS: 87.07 months *vs*. not reached, *p* < 0.001, **Figure 5G**). Additionally, stratification analysis further exhibited robustness of the FARG signature in prognosis prediction, which had the potential to predict OS for early-stage BC patients (**Figure 5H**). As shown, high-risk patients always had worse OS regardless of their histological or clinical feature. As aforementioned, there was no significant difference in OS of patients with long distant metastasis between high- and low-risk groups, probably owing to the limited patient number.

### Construction of a FARG signature-based nomogram

**Figure 6:**
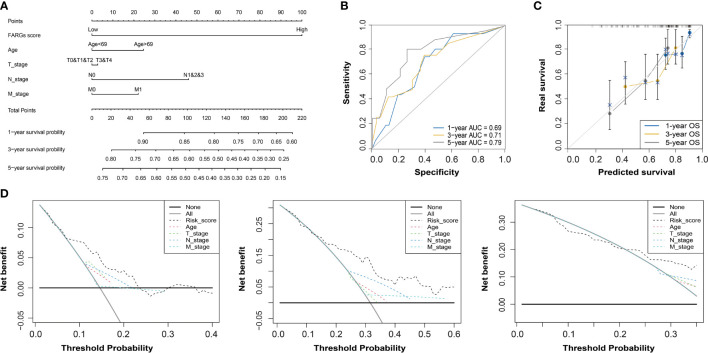
The construction and evaluation of a FARG-based nomogram. **(A)** Based on the independent prognostic factor of the FARG score together with clinical features of diagnosis age, and T, N, M stages, a novel nomogram was constructed. **(B)** The receiver operating characteristic curve analysis for evaluation of the FARG-based nomogram in prognosis prediction. **(C)** The calibration plots further estimated the ability of the FARG-based nomogram in prognosis prediction. **(D)** The decision curve analysis for the evaluation of the FARG-based nomogram in prognosis prediction.

Subsequently, the univariate Cox regression analysis further revealed that FARG score, together with clinical features including diagnosis age, T, N, M stage, and neoplasm disease stage, was all negatively correlated with OS of BC patients (*p* < 0.05, **Table 2**). Of note, only FARG score was an independent prognostic factor *via* the multivariate Cox regression analysis (*p* < 0.01, **Table 2**). Based on the FARG score concurrently combined with diagnosis age and TNM stage, the FARG signature-based nomogram (C-index: 0.70, 95% confidence interval: 0.61–0.79) was ultimately constructed (**Figure 6A**). The AUC values of nomogram for 1-, 3-, and 5-year OS were 0.69, 0.71, and 0.79, respectively (**Figure 6B**). Moreover, the calibration curves also showed good consistency between actual and predictive clinical outcomes by nomogram (**Figure 6C**). The DC analysis (**Figure 6D**) further revealed that the novel constructed nomogram was a robust prognosis prediction model compared to the traditional histological or clinical features.

**Table 2:** Univariate and multivariate Cox regression analysis of the FARG signature and clinical features and OS of BC patients from TCGA.

Variable	Univariate			Multivariate		
	HR	95% CI	*p*-value	HR	95% CI	*p*-value
FARG score						
High/Low	2.12	1.46–3.10	<0.01	3.89	1.64–9.23	<0.01
Age						
>69/<69	1.44	1.00–2.07	<0.05	1.47	0.76–2.83	0.25
Gender						
Male/Female	0.79	0.53–1.16	=0.23	0.50	0.24–1.04	0.06
T stage						
T3,4/T0,1,2	1.74	1.12–2.70	=0.01	1.02	0.41–2.07	0.84
N stage						
N1,2,3/N0	2.84	1.91–4.22	<0.01	1.48	0.71–3.05	0.29
M stage						
M1/M0	4.22	1.78–10.01	<0.01	1.49	0.41–5.37	0.54
Neoplasm stage						
III,IV/I,II	2.69	1.87–3.88	<0.01	1.39	0.67–3.22	0.45
*FGFR3* status						
Mut/WT	0.74	0.42–1.29	0.29	0.80	0.26–2.42	0.69
*FGFR3* expression						
High/Low	1.04	0.98–1.11	0.22	1.15	1.00–1.33	0.07

OS, Overall survival; HR, Hazard ratio; CI, Confidence interval; Mut, Mutated; WT, Wild-type.

### Biological functions associated with FARG signature

**Figure 7:**
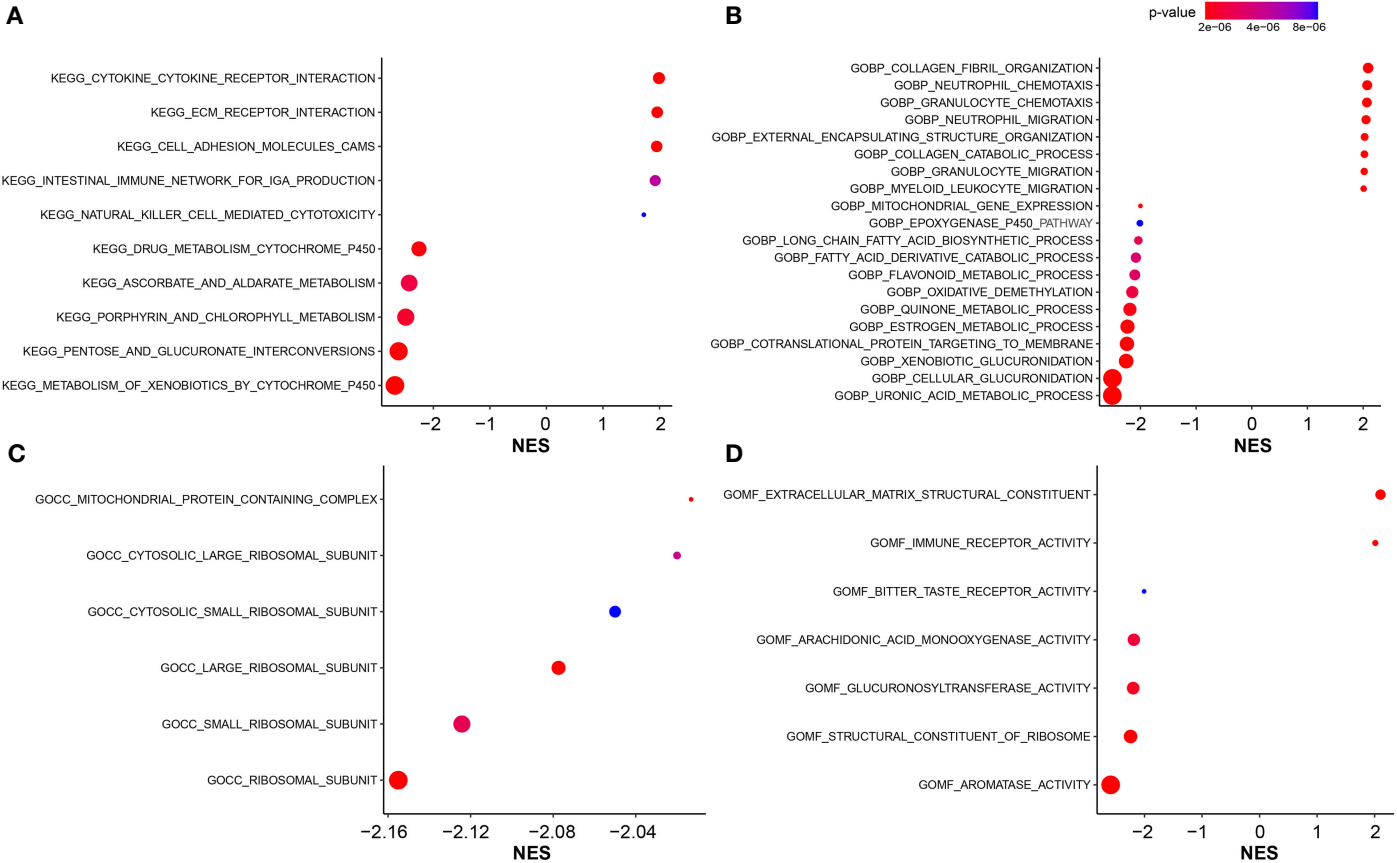
The functional enrichment analysis. **(A)** The KEGG pathway enrichment analysis for identification of biological functions associated with FARG signature. **(B)** The GO enrichment analysis for identification of biological processes (GOBP) associated with FARG signature. **(C)** The GO enrichment analysis for identification of cellular components (GOCC) associated with FARG signature. **(D)** The GO enrichment analysis for identification of molecular functions (GOMF) associated with FARG signature.

GO and KEGG enrichment analyses were utilized to illustrate the biological functions and pathways related to the FARG signature. Interestingly, KEGG pathway analysis revealed that the genes were mainly enriched in the metabolism- and immune-related signaling pathways. A series of KEGG pathways, such as xenobiotics, pentose and glucuronate interconversions, and porphyrin and chlorophyll metabolisms, were highly enriched in the low-risk group (**Figure 7A**), while the high-risk group had the enrichment of cytokine–cytokine receptor interaction, extracellular matrix (ECM) receptor interaction, cell adhesion molecules, intestinal immune network for IgA production, and natural killer cell-mediated cytotoxicity (**Figure 7A**). In addition, GO-BP enrichment analysis exhibited the enrichment of uronic acid metabolic, cellular glucuronidation, and xenobiotic glucuronidation in the low-risk group, but collagen fibril organization, neutrophil chemotaxis, and granulocyte chemotaxis in the high-risk group (**Figure 7B**). For the GO-CC category, related ribosomal subunits and mitochondrial protein-containing complex were significantly enriched in the low-risk group (**Figure 7C**). Furthermore, for category MF, the main functions of ECM structural constituent and immune receptor activity were enriched in the high-risk group, whereas aromatase activity, structural constituent of ribosome, and glucuronosyltransferase activity were abundantly enriched in the low-risk group (**Figure 7D**).

### Genomic alteration enrichment analysis

**Figure 8:**
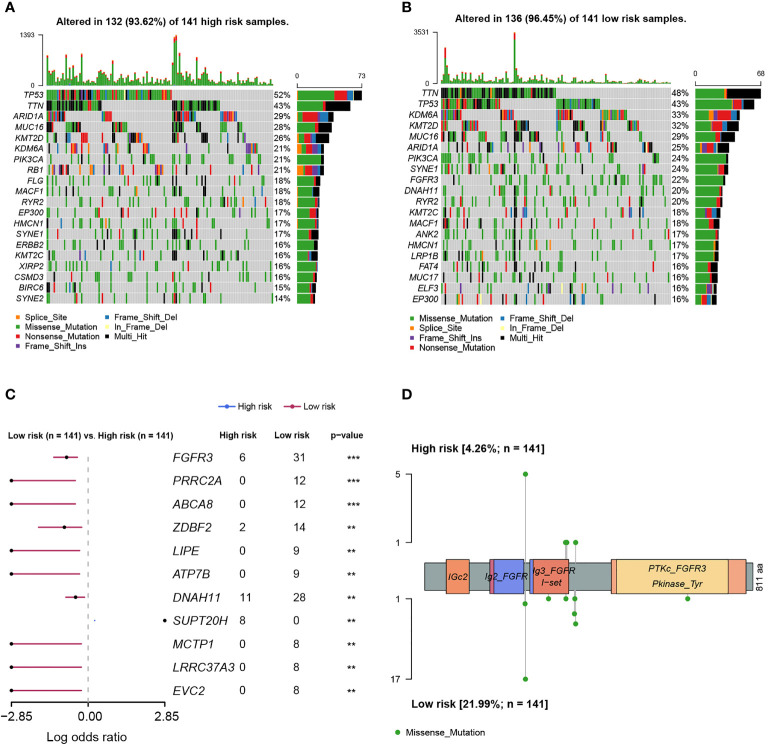
The differentiation analysis of genomic alteration landscapes between high- and low-risk groups. **(A)** The oncoplot demonstrated the genomic alteration profile of high-risk samples. **(B)** The oncoplot demonstrated the genomic alteration profile of low-risk samples. **(C)** The genomic alteration enrichment analysis between high- and low-risk groups. **(D)** The lollipop plot demonstrated the alteration sites of high- and low-risk samples. ** indicated *p* < 0.01, *** indicated *p* < 0.001.

As was known, transcriptomic dysfunctions, leading to tumor development and progression, were usually attributed to genomic alterations. Initially, we found that there was no statistically significant difference in mutation counts between high- and low-risk groups (**Supplementary Figure 4**). Subsequently, enrichment analysis was conducted to investigate the difference in genomic alterations between these two groups. The oncoprint plot exhibited that the top 20 most frequently altered genes between two groups were noticeably distinct (**Figures 8A, B**). Especially for the *FGFR3* gene, its alteration frequency was extremely higher in the low-risk group (21.99% *vs*. 4.26%, *p* < 0.001, **Figure 8C**). Underlying the FARG signature, *SUPT20H* alterations were found to be only prevalent in the high-risk group (5.67% *vs*. 0.00%, *p* < 0.01, **Figure 8C**); on the contrary, the alteration frequencies of *PRRC2A* (8.51% *vs*. 0.00%, *p* < 0.001), *ABCA8* (8.51% *vs*. 0.00%, *p* < 0.001), *ZDBF2* (9.93% *vs*. 1.42%, *p* < 0.01), and other genes were higher in the low-risk group. Intriguingly, all the *FGFR3* alterations were missense (**Figure 8D**); furthermore, c.746C>G (p.Ser249Cys) was highlighted and more prevalent in the low-risk group (*p* < 0.05); however, the Kaplan–Meier curve analysis revealed that there was no statistically significant difference in the OS between groups with *FGFR3* mutations dominated by c.746C>G (p.Ser249Cys) or other sites, and without *FGFR3* mutations (*p* > 0.05, **Supplementary Figure 5**).

### TME and immunotherapy response correlated with FARG signature

**Figure 9:**
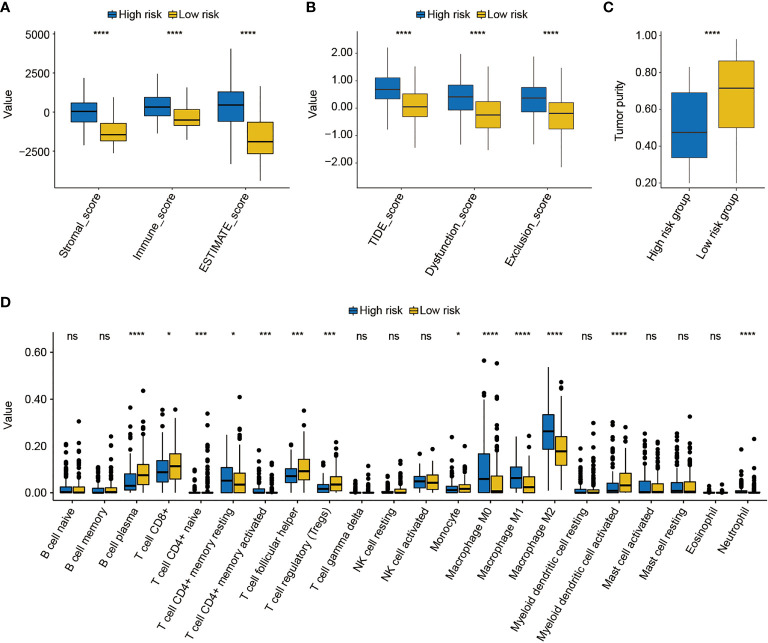
The analysis of the tumor environment between high- and low-risk groups. **(A)** Based on the ESTIMATE algorithm, it was found that low-risk BC patients had lower stromal, immune, and ESTIMATE scores. **(B)** Further analysis showed that low-risk BC patients had lower dysfunction, exclusion, and T-cell dysfunction and exclusion (TIDE) scores. **(C)** The evaluation of tumor purity of high- and low-risk BC patients. **(D)** The estimate of 22 common tumor-infiltrated lymphocytes between high- and low-risk groups. * indicated *p* < 0.05, *** indicated *p* < 0.001, **** indicated *p* < 0.0001, ns indicated no statistically significant difference.

Subsequently, the differed features in TME between high- and low-risk groups were evaluated, and it was found that BC patients in the high-risk group had significantly higher stromal, immune, and ESTIMATE scores than patients in the low-risk group (*p* < 0.0001, **Figure 9A**). Moreover, the high-risk group was also closely correlated with the higher T-cell dysfunction, T-cell exclusion, and TIDE scores (*p* < 0.0001, **Figure 9B**). The evaluation of tumor purity further showed that the low-risk group had the higher tumor purity (*p* < 0.0001, **Figure 9C**), whereas it was observed that there was no statistically significant difference in the number of clonal and subclonal neoantigens between high- and low-risk groups (*p* > 0.05, **Supplementary Figure 6**). Furthermore, the analysis of 22 kinds of TILs revealed that plasma B cells, CD8+ T cells, CD4+ naive T cells, helper follicular T cells, regulatory T cells (Tregs), monocytes, and activated myeloid dendritic cells were significantly enriched in the low-risk group. However, the infiltrated levels of resting CD4+ memory T cells, activated CD4+ memory T cells, M0 macrophage, M1 macrophage, M2 macrophage, and neutrophils were higher in the high-risk group (*p* < 0.05, **Figure 9D**).

**Figure 10:**
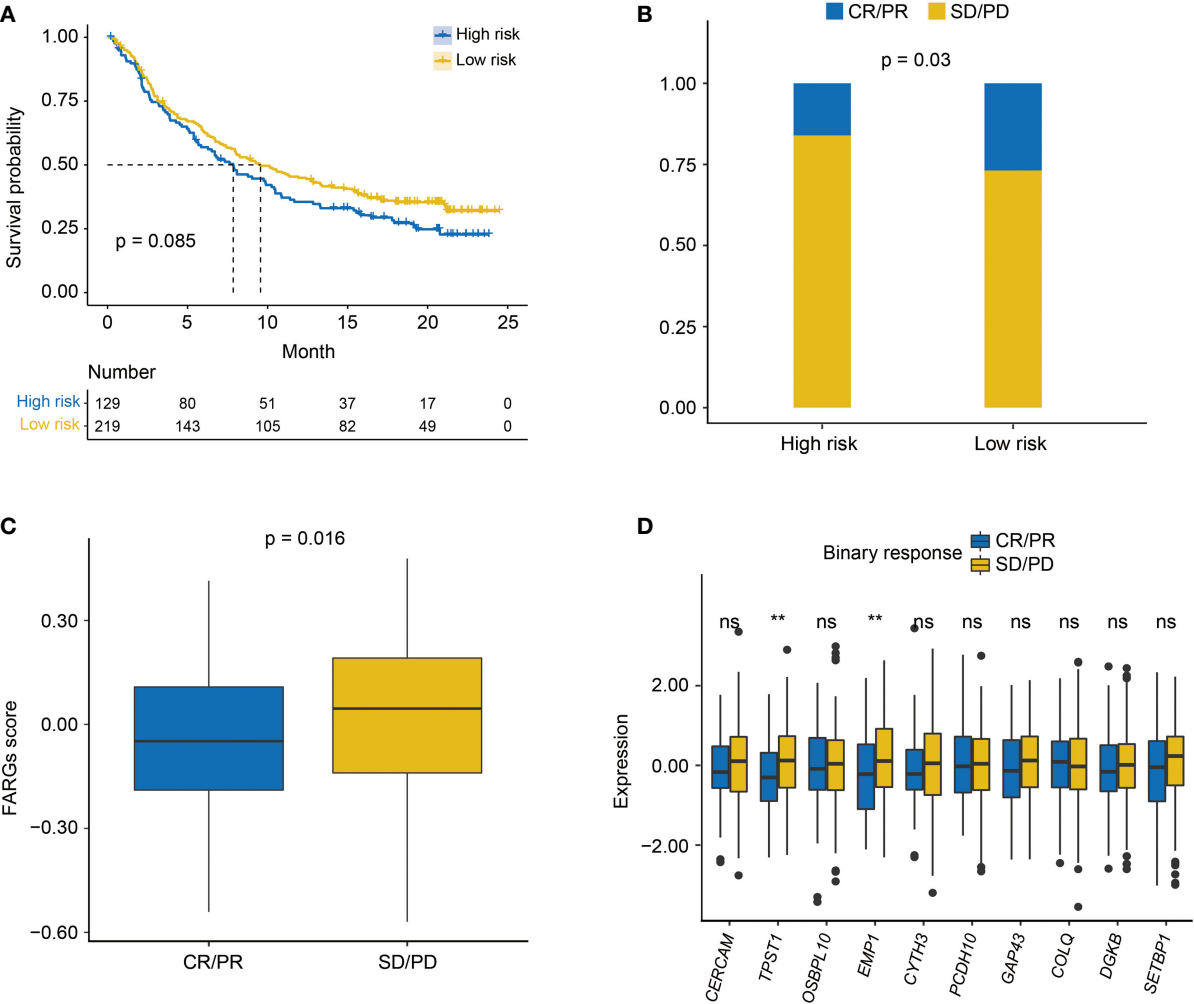
The evaluation of immunotherapy response by the FARG signature in the IMvigor210 cohort. **(A)** The Kaplan–Meier curve analysis between high- and low-risk groups in the IMvigor210 cohort. **(B)** The proportion of BC patients made complete/partial response (CR/PR) or kept a stable/progressive disease (SD/PD) in high- and low-risk groups. **(C)** The comparison of FARG score between patients making complete/partial response (CR/PR) and those who kept a stable/progressive disease (SD/PD). **(D)** The lower expression of *TPST1* and *EMP1* was significantly associated with the better immunotherapy response of anti-PD-L1/PD-1 treatment in the analysis of the IMvigor210 cohort. ** indicated *p* < 0.01, ns indicated no statistically significant difference.

As found above, there was a significant difference in TME between high- and low-risk groups. Thus, the IMvigor210 cohort was then employed to investigate the predictive significance of FARG signature for immunotherapy response. Survival analysis revealed that BC patients with lower FARG scores presented with relatively better clinical outcomes (median OS: 9.56 months *vs*. 7.85 months, *p* = 0.085, **Figure 10A**). Remarkably, it was found that there was a higher proportion (26.88% *vs*. 16.07%, *p* = 0.03, **Figure 10B**) of BC patients in the low-risk group who responded to the immunotherapy. Moreover, comparison analysis of FARG scores between CR/PR and PD/SD groups exhibited that BC patients in the CR/PR group had a significantly lower FARG score (*p* = 0.016, **Figure 10C**), indicating that FARG signature could serve as an indicator in the treatment response of immunotherapy. Of note, it was further found that lower gene expression levels of *TPST1* and *EMP1*, two genes from the FARG signature, were correlated with the immunotherapy response (*p* < 0.01, **Figure 10D**) among BC patients from the IMvigor210 cohort, whereas *FGFR3* expression was not found to be significantly associated with immunotherapy response in the IMvigor210 cohort (*p* = 0.24, **Supplementary Figure 7**).

### Evaluation of chemotherapy response

**Figure 11:**
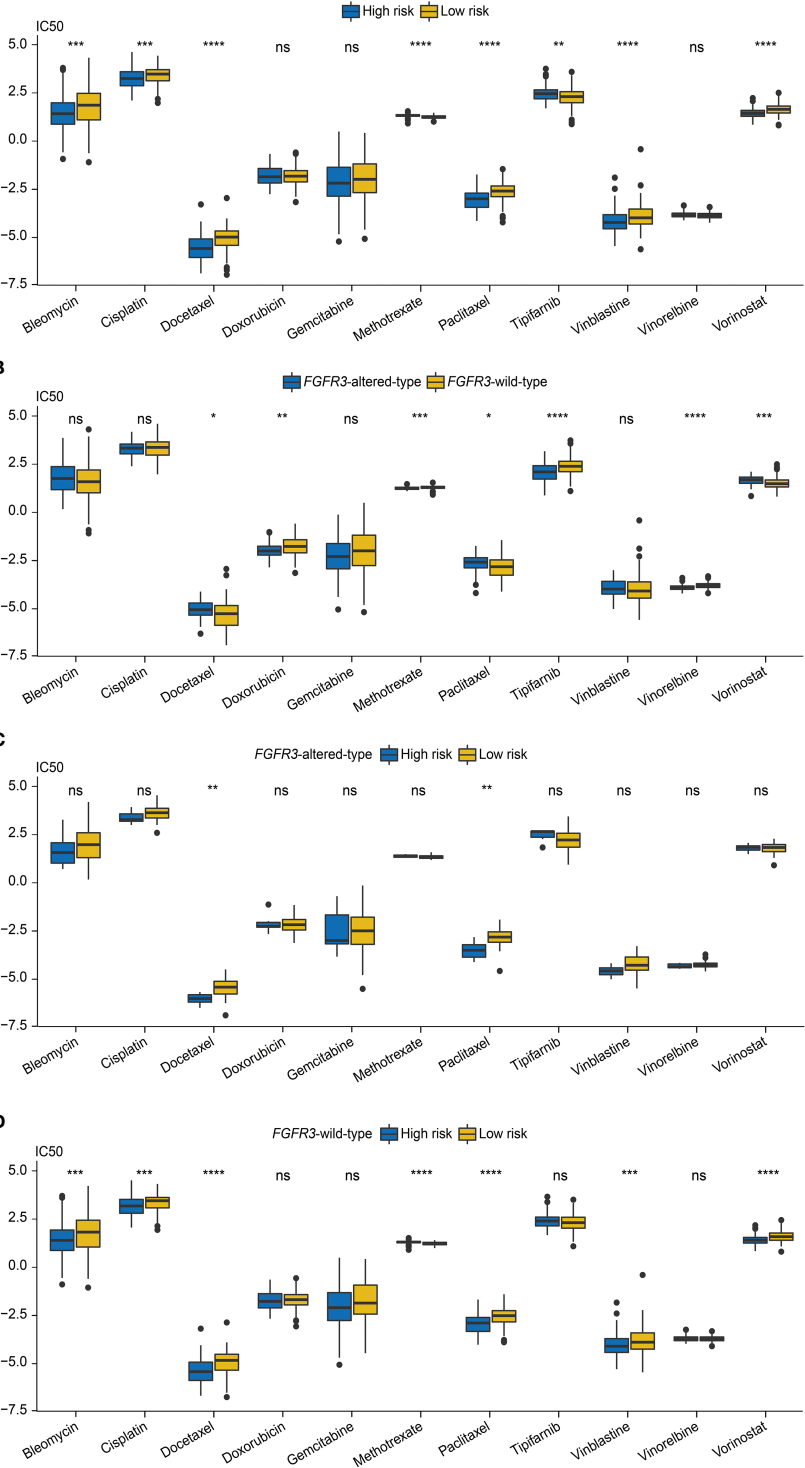
The evaluation of chemotherapy response based on the analysis of Genomics of Drug Sensitivity in Cancer (GDSC). **(A)** The comparison of IC_50_ values between high- and low-risk groups. **(B)** The comparison of IC_50_ values between patients with *FGFR3* alterations or with wild-type *FGFR3*. **(C)** The comparison of IC_50_ values between high- and low-risk groups of patients with *FGFR3* alterations. **(D)** The comparison of IC_50_ values between high- and low-risk groups of patients with wild-type *FGFR3*. * indicated *p* < 0.05, ** indicated *p* < 0.01, *** indicated *p* < 0.001, **** indicated *p* < 0.0001, ns indicated no statistically significant difference.

Next, the role of the FARG signature in predicting chemotherapy response was examined. It was found that MIBC patients treated with MVAC frontline chemotherapy from the GSE48276 dataset had significantly declined FARG scores (*p* < 0.001, **Supplementary Figure 8**). Additionally, chemo-drug sensitivity analysis *via* the GDSC database further exhibited that the IC_50_ of bleomycin, cisplatin, docetaxel, paclitaxel, vinblastine, and vorinostat was significantly lower in the high-risk group, whereas the IC_50_ of methotrexate and tipifarnib was lower in the low-risk group (**Figure 11A**).

Subsequently, the chemotherapy response was further estimated for *FGFR3*-altered or wild-type BC patients *via* the analysis of the GDSC database. First, it was observed that the IC_50_ values of doxorubicin, methotrexate, tipifarnib, and vinorelbine were lower for patients with altered *FGFR3*, but there were relatively lower IC_50_ values of docetaxel, paclitaxel, and vorinostat for patients with wild-type *FGFR3* (**Figure 11B**). For patients with altered *FGFR3*, sensitivities to these investigated chemo-drugs were almost equivalent between high- and low-risk groups, except for docetaxel and paclitaxel, of which IC_50_ values significantly declined in the high-risk group (**Figure 11C**). Nevertheless, for patients with wild-type *FGFR3*, the IC_50_ of bleomycin, cisplatin, docetaxel, paclitaxel, vinblastine, and vorinostat was noticeably decreased in the high-risk group, while there was only methotrexate with the lower IC_50_ value in the low-risk group (**Figure 11D**).

## Discussion

In urothelial carcinoma, *FGFR3* alterations were found to be an early event (28), and were further identified as a prognostic factor in BC patients (29). Currently, it is suggested that *FGFR3* alterations are highly correlated with BC prognosis as well as regarded as the only US FDA-approved biomarker for precision medicine in BC (30, 31). As described, a lot of studies have revealed the vital role of *FGFR3* alterations in BC; however, the unique transcriptomic profile closely linked to *FGFR3* alterations remains nearly unknown. In the present study, WGCNA identified a comprehensive *FGFR3* alteration-related transcriptomic profile, which was further used to establish a novel FARG signature. Not only did this FARG signature perform well in prognosis prediction, it also had the potential to be an effective indicator for the response prediction of immunotherapy and chemotherapy. Furthermore, this signature was an independent prognostic factor for BC patients and a novel signature-based prognostic nomogram exhibited better performance in prognosis prediction.

Among these 11 genes involved in the FARG signature, the expression levels of 9 genes, namely, *CERCAM*, *TPST1*, *OSBPL10*, *EMP1*, *CYTH3*, *PCDH10*, *GAP43*, *DGKB*, and *SETBP1*, were found to be negatively correlated with survival of BC patients. In a previous study, both *in vivo* and *in vitro* experiments revealed that *CERCAM*, as an oncogenic gene, was markedly elevated in BC tissue samples, the overexpression of which significantly enhanced the viabilities and invasions of BC cells (32). Except for *CERCAM*, the prognostic function of 8 other genes was first identified in BC, and their overexpression was suggested to promote the development and progression of BC. Notably, this was the first study finding in which the expression level of non-coding RNA gene *NCRNA00201* was positively correlated with clinical outcomes of BC patients. Further study is merited to provide more deep insights into how they involve or regulate the development of BC. Importantly, it was further found *via* the analysis of the IMvigor210 cohort that *TPST1* and *EMP1* could successfully predict the response of immunotherapy. Moreover, BC patients from the IMvigor210 cohort, who had objective responses to anti-PD-L1 treatment, had remarkably decreased expressions of *TPST1* and *EMP1*, the suppressed expressions of which might improve the treatment response of the monoclonal antibody atezolizumab. A previous study analyzing the TCGA cohort also demonstrated that elevated expression of *EMP1* was correlated with worse prognosis of BC patients and could significantly affect the abundance of TILs (33). Of note, it was first reported in the present study that *TPST1* gene expression could exert influence on TME and even be correlated with immunotherapy in BC. Overall, these results found in the present study needed further investigations and experimental verifications.

According to the results of biological function enrichment, it was noticeably found *via* KEGG enrichment analysis that the markedly enriched pathways were almost associated with metabolisms, immunity, and inflammation, indicating that FARG signature and related genes were highly correlated with the regulation of immune or inflammation-related signaling pathways, which would directly influence the TME. Moreover, the biological process category *via* GO enrichment also displayed that cancer metabolisms and TME enormously contributed to BC progression as well (34, 35). The cellular component category *via* GO enrichment first revealed that the aberrant regulation of predominantly enriched ribosomal subunits were correlated with BC progression, although there were some studies that illustrated that perturbation of ribosomal biogenesis (36) or ribosomal protein expression (37) could promote cancer or cancer metastasis, respectively. In addition, molecular function category *via* GO enrichment further exhibited the important role of ribosome or ribosomal-related structural constituent in BC progression. Furthermore, in the present study, aromatase and glucuronosyltransferase activities were also enriched. As was known, epirubicin, predominantly metabolized by the glucuronosyltransferase, has been clinically applied for breast cancer treatment (38); moreover, aromatase inhibitor treatment mainly functions for breast cancer by decreasing estrogen production (39). However, the role of glucuronosyltransferase and aromatase in BC remained to be fully elucidated, whether therapies targeting glucuronosyltransferase and aromatase in BC were controversial. Underlying the FARG signature, more immune- and stroma-related biological activities were identified to play a vital role in the progression of BC; thus, it seemed that TME remodeling was more preferred for the treatment of BC; meanwhile, TME was highly correlated with immunotherapy response.

As expected, *FGFR3* alterations were found to be more prevalent in the low-risk group. Consistent with previously described results, *FGFR3* alteration frequency was positively correlated with prognosis of BC patients. Underlying the FARG signature, it was first identified in the present study that among all *FGFR3* alterations, c.746C>G (p.Ser249Cys) was significantly more prevalent in the low-risk group; however, it was found in the present study that this mutation site was not significantly correlated with OS of BC patients, whereas there were few studies currently focusing on the *FGFR3* mutation site c.746C>G (p.Ser249Cys), except that FGFR antagonists could inhibit TCC97-7-type BC cells that carried the c.746C>G (p.Ser249Cys) of *FGFR3* mutation (40). In the clinic, there was a case report describing that an advanced upper urinary tract urothelial carcinoma (UTUC) patient with *FGFR3* c.746C>G (p.Ser249Cys) could make a complete response to immunotherapy with the monoclonal antibody pembrolizumab (41), but whether *FGFR3* c.746C>G (p.Ser249Cys) might influence the treatment response of BC patients was totally unknown and worth further investigation. Indeed, we found in the present study that the *FGFR3* mutation site c.746C>G (p.Ser249Cys) was significantly prevalent in the low-risk group; of note, low-risk patients in the IMvigor210 cohort made significantly better responses to immunotherapy, but the direct association between c.746C>G (p.Ser249Cys) and immunotherapy response was fully unclear. In addition, underlying the FARG signature, it was first observed that *PRRC2A*, *ABCA8*, *ZDBF2*, *LIPE*, *ATP7B*, *DNAH11*, *MCTP1*, *LRRC37A3*, and *EVC2* gene alterations were correlated with prolonged OS of BC patients, but altered *SUPT20H* might be associated with shortened OS.

As known, the ESTIMATE method facilitated the prediction of tumor purity by assessing stromal cells and immune cells in tumor tissues (22), and TIDE score including T-cell dysfunction and exclusion scores not only correlated with patient survival under immunotherapy but could also predict the immunotherapy response by immune checkpoint blockade (23). Of note, in the present study, it was revealed that the FARG score was positively correlated with ESTIMATE score, which was negatively correlated with tumor purity. Similarly, the FARG score was also positively correlated with TIDE score, indicating that BC patients with lower FARG score might benefit more from the immunotherapy treatment (23). The evaluation of 22 TILs further manifested that plasma B cells, CD8+ T cells, CD4+ naive T cells, and helper follicular T cells were abundantly enriched in the low-risk group, and these TILs could greatly improve immune response as well as immunotherapy response (42). Notably, it was also identified that the low-risk group had the enrichment of regulatory T cells (Tregs), which could inhibit effective tumor immunity (43) and be associated with poorer clinical outcomes in BC (44). Controversially, a growing body of evidence showed that regulatory T cells (Tregs) were a key regulator of anti-tumor response in BC (42), especially for Bacillus Calmette–Guerin (BCG) immunotherapy (45). Furthermore, BCG immunotherapy could also increasingly release the monocyte cytolytic factor, which could improve the anti-tumor activity (46). The activated myeloid dendritic cells connected the innate and adoptive immune system mechanisms, capable of delivering tumor antigen and stimulating immune response (47). Also, both monocyte and activated myeloid dendritic cells were markedly enriched in the low-risk group. From multiple perspectives, it could be inferred that BC patients in the low-risk group were more likely to make significant responses to immunotherapy.

Furthermore, as found in the IMvigor210 cohort, BC patients in the CR/PR group had a significantly lower FARG score. The FARG signature helped distinguish BC patients who would be more suitable to receive immunotherapy by the current immune checkpoint blockade. Overall, this signature had the potential to become an effective indicator to predict the immunotherapy response. In addition, it was identified in the present study that MIBC patients had a noticeably decreased FARG score after receiving MVAC frontline chemotherapy treatment, indicating that the FARG signature could probably further predict the treatment efficacy of MVAC frontline chemotherapy, which has been the standard treatment for MIBC patients after receiving transurethral resection (48), whereas these needed further clinical trials for validation. By analyzing the GDSC database, the FARG signature predicted that irrespective of carrying *FGFR3* alterations or not, BC patients with a lower FARG score were likely to be more sensitive to methotrexate and tipifarnib, whereas those with a higher FARG score seemed to have higher sensitivities to bleomycin, cisplatin, docetaxel, paclitaxel, vinblastine, and vorinostat. Of note, the chemo-drug sensitivity of patients from high- and low-risk groups changed if *FGFR3* status was taken into consideration. Therefore, it was highly recommended by clinicians that the *FGFR3* status and the unique transcriptomic profile determined by *FGFR3* alterations should be considered when making treatment selections for BC patients. In brief, this FARG signature based on a comprehensive *FGFR3* alteration-related transcriptomic profile exhibited its good and reliable ability in prognosis prediction, and would promote personalized treatment and precision medicine.

## Conclusions

This was the first study to systematically investigate the unique transcriptomic profile determined by *FGFR3* alterations, and the *FGFR3* alteration-related transcriptomic characterization is implicated with biological activities, molecular features, and tumor immune infiltration of BC. Based on the *FGFR3* alteration-related transcriptome, a novel FARG signature was constructed and found to have good performance predicting prognosis and treatment responses, especially immunotherapy responses.

## Data availability statement

In the present study, publicly available datasets, including training group of TCGA-BC cohort (MSK/TCGA, 2020, https://www.cbioportal.org/study/summary?id=blca_msk_tcga_2020) and validation groups of GSE13507, GSE31684, GSE32548, GSE48075 datasets from GEO database (http://www.ncbi.nlm.nih.gov/geo/), were analyzed for construction of prognostic signature. The IMvigor210 cohort of BC patients receiving immunotherapy was retrieved from the following website: https://clinicaltrials.gov/ct2/show/NCT02108652. The GSE48276 dataset was downloaded from the GEO for chemotherapy response evaluation.

## Author contributions

TX and BY proposed this work and designed the study; WX, YZ, and XL collected the raw data. TX, WX, YZ, XL, and HC conducted data analysis. TX, WX, and XL contributed to figure visualization; TX, WX, ZX, QZ, and BY drafted the manuscript; TX and BY revised the manuscript; All authors contributed to the article and approved the submitted version.

## Funding

This study was supported by the Department of Urology, Jiangsu Institute of Cancer Research, the Affiliated Cancer Hospital (Jiangsu Cancer Hospital) of Nanjing Medical University and the Wu Jie-Ping Medical Foundation (Project No. 320.6750.2020-10-113).

## Acknowledgments

We appreciate the help of all colleagues involved in this study and the financial support from the Affiliated Cancer Hospital (Jiangsu Cancer Hospital) of Nanjing Medical University, Nanjing, Jiangsu Province, People’s Republic of China. Moreover, we thank the technical assistance provided by the Lifehealthcare Clinical Laboratories, Hangzhou, Zhejiang Province, People’s Republic of China.

## Conflict of interest

The authors declare that the research was conducted in the absence of any commercial or financial relationships that could be construed as a potential conflict of interest.

## Publisher’s note

All claims expressed in this article are solely those of the authors and do not necessarily represent those of their affiliated organizations, or those of the publisher, the editors and the reviewers. Any product that may be evaluated in this article, or claim that may be made by its manufacturer, is not guaranteed or endorsed by the publisher.
