# Supplementary figures and images for: Comprehensive FGFR3 alteration-related transcriptomic characterization is involved in immune infiltration and correlated with prognosis and immunotherapy response of bladder cancer
Source: Front Immunol. 2022 Jul 26;13:931906. doi: 10.3389/fimmu.2022.931906 (PMC9360490; doi:10.3389/fimmu.2022.931906)

FGFR3mut FGFR3wt

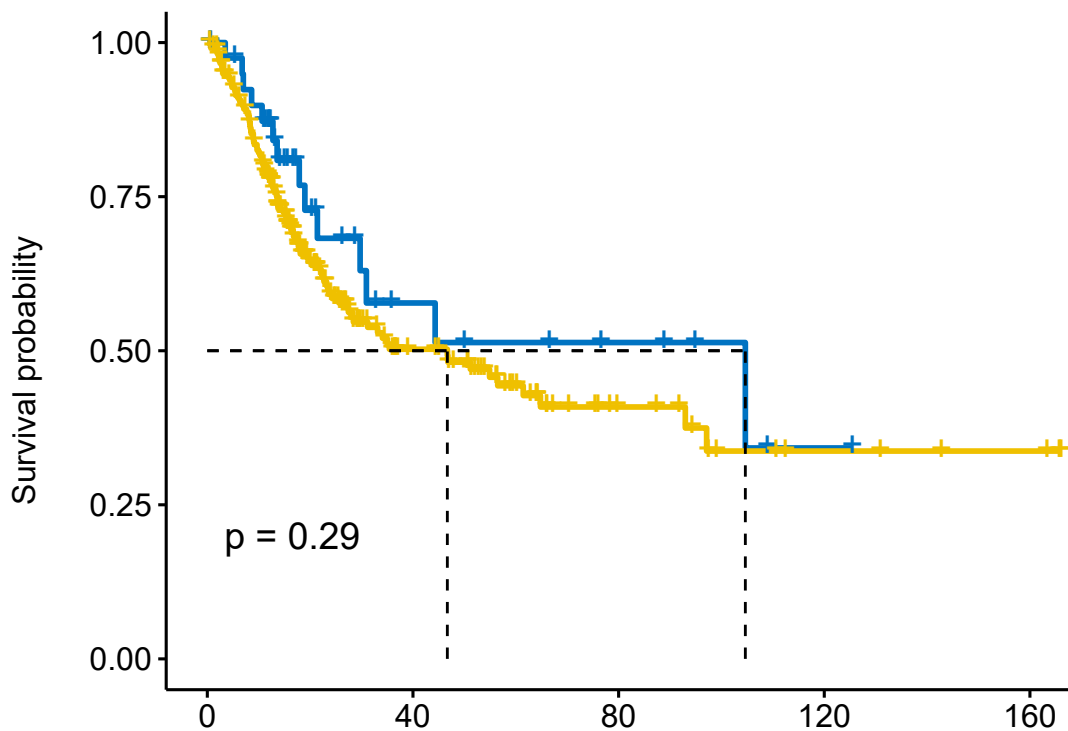

Number at risk

|          |      |    |    |     |     |
|----------|------|----|----|-----|-----|
| FGFR3mut | 42   | 9  | 5  | 1   | 0   |
| FGFR3wt  | 240  | 50 | 14 | 5   | 3   |
|          | 0    | 40 | 80 | 120 | 160 |
|          | Time |    |    |     |     |

Supplement: Supplementary file 1 [file Image_1.pdf]

A

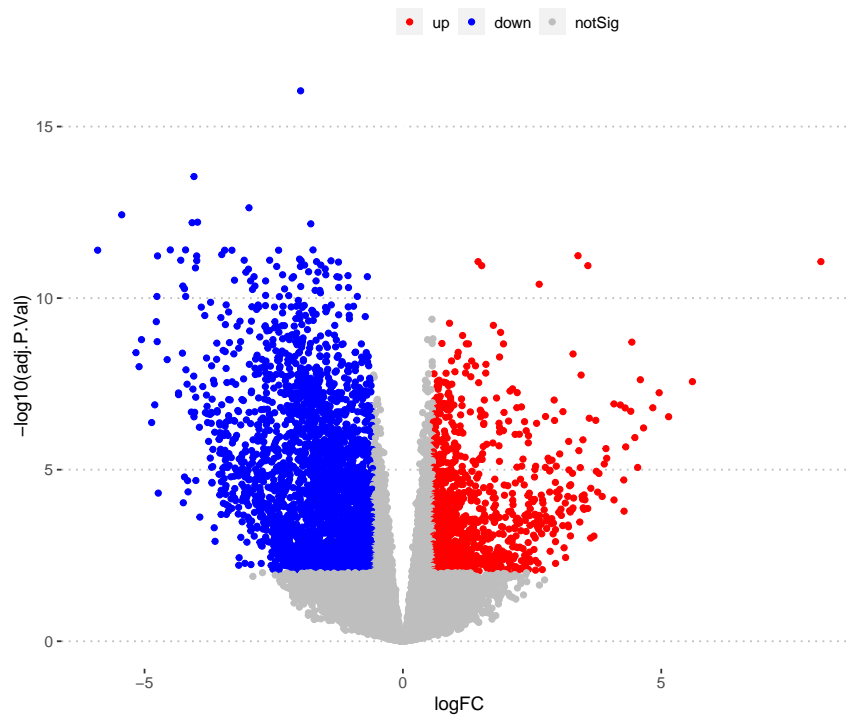

B

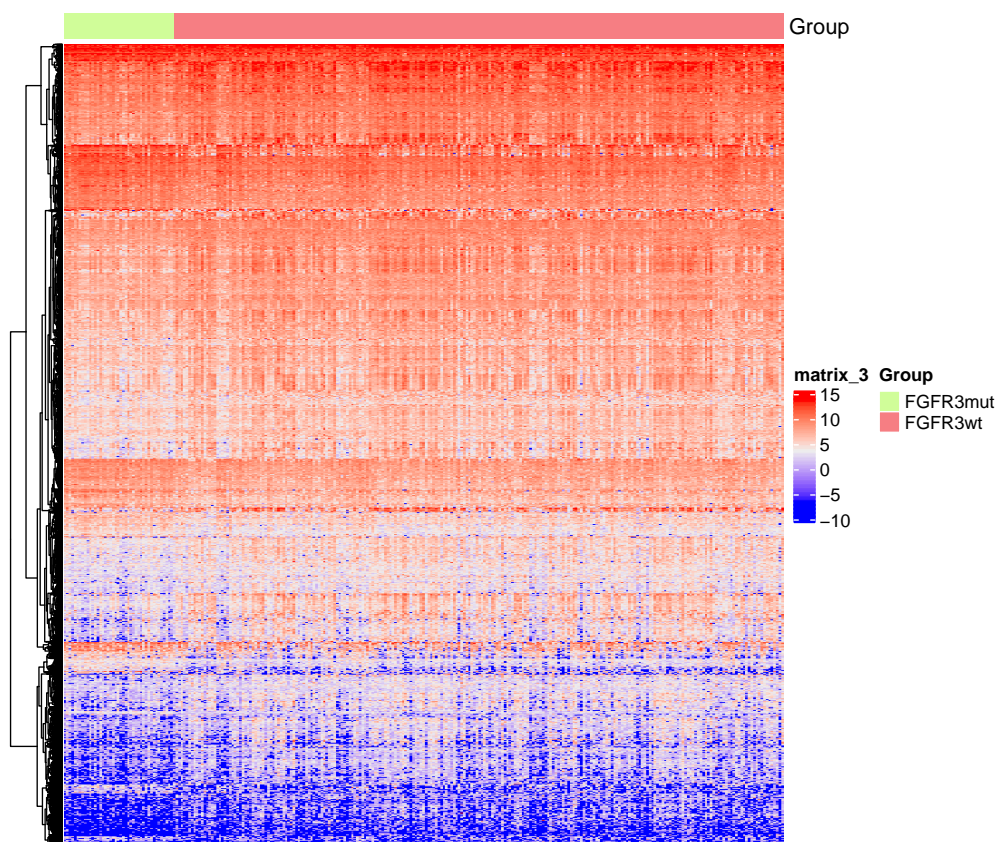

Supplement: Supplementary file 2 [file Image_2.pdf]

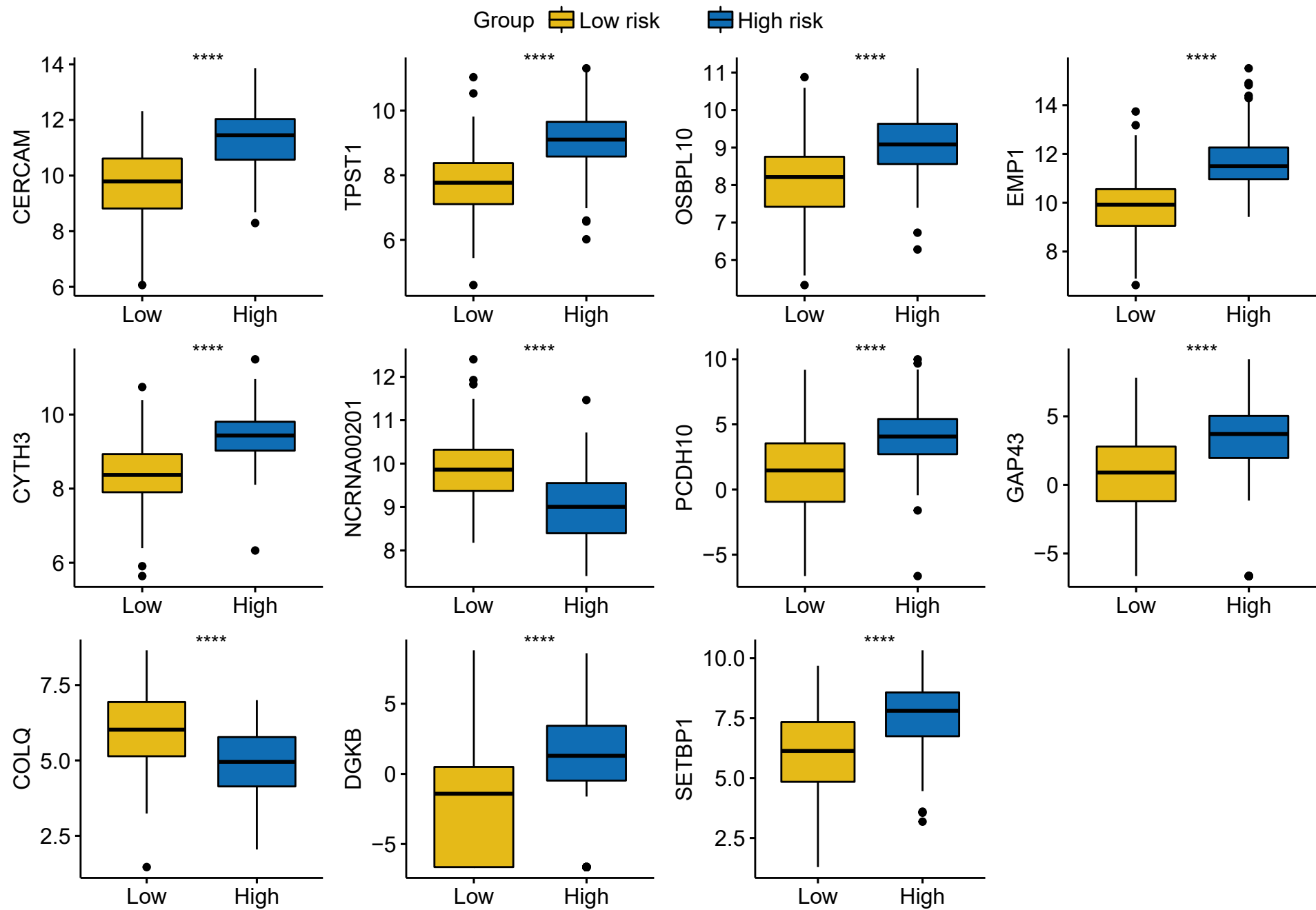

Supplement: Supplementary file 3 [file Image_3.pdf]

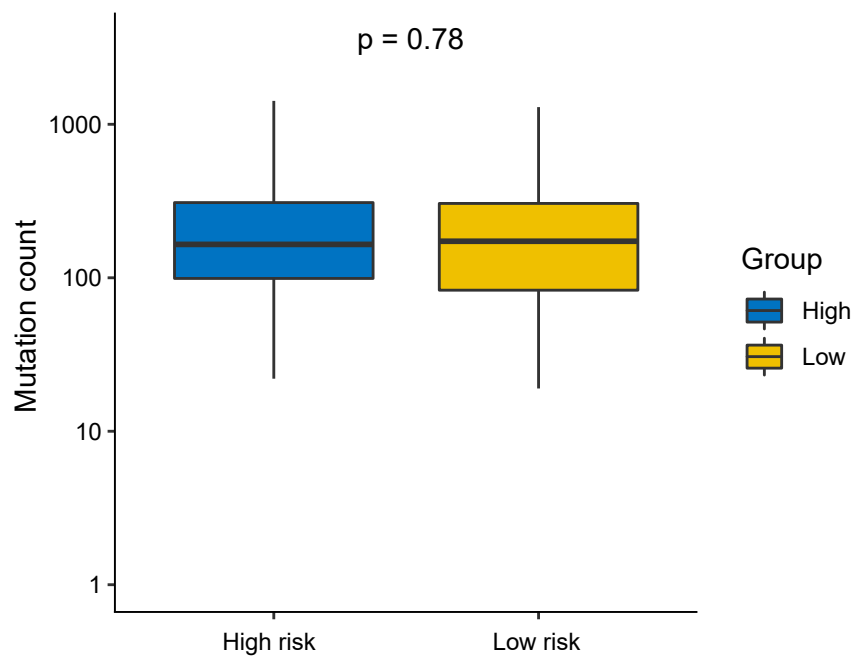

Supplement: Supplementary file 4 [file Image_4.pdf]

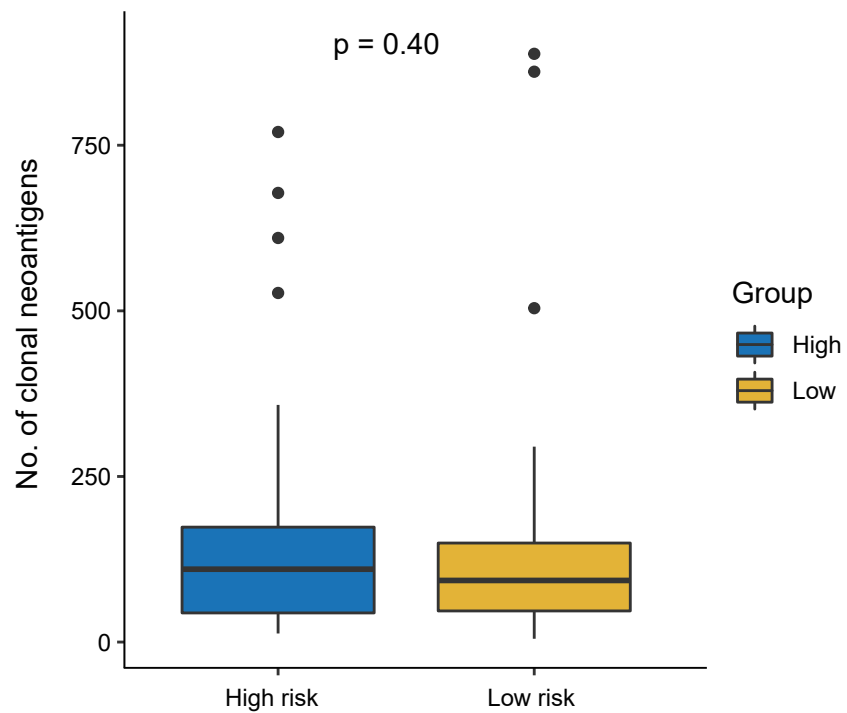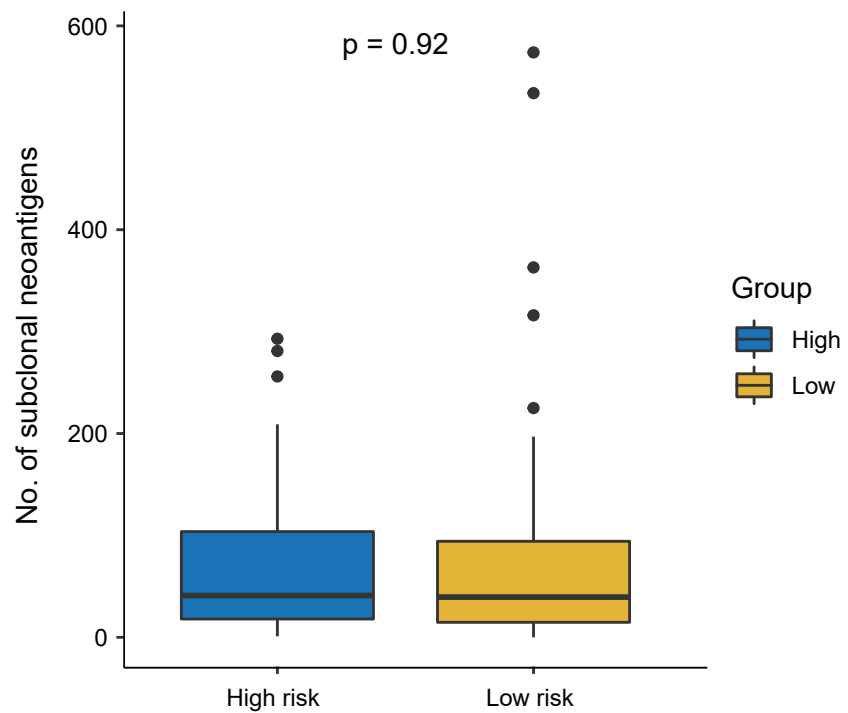

Supplement: Supplementary file 6 [file Image_6.pdf]

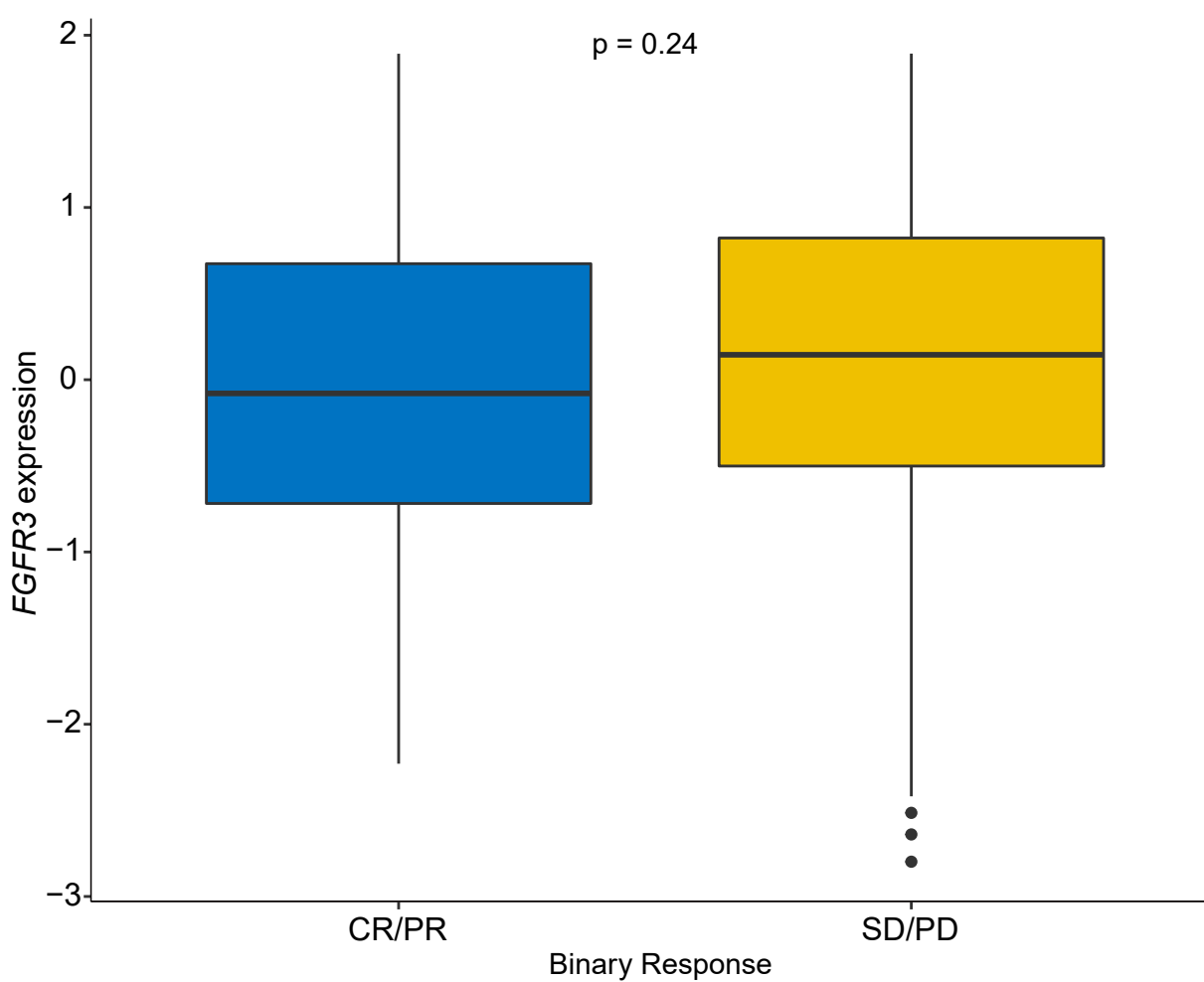

Supplement: Supplementary file 7 [file Image_7.pdf]

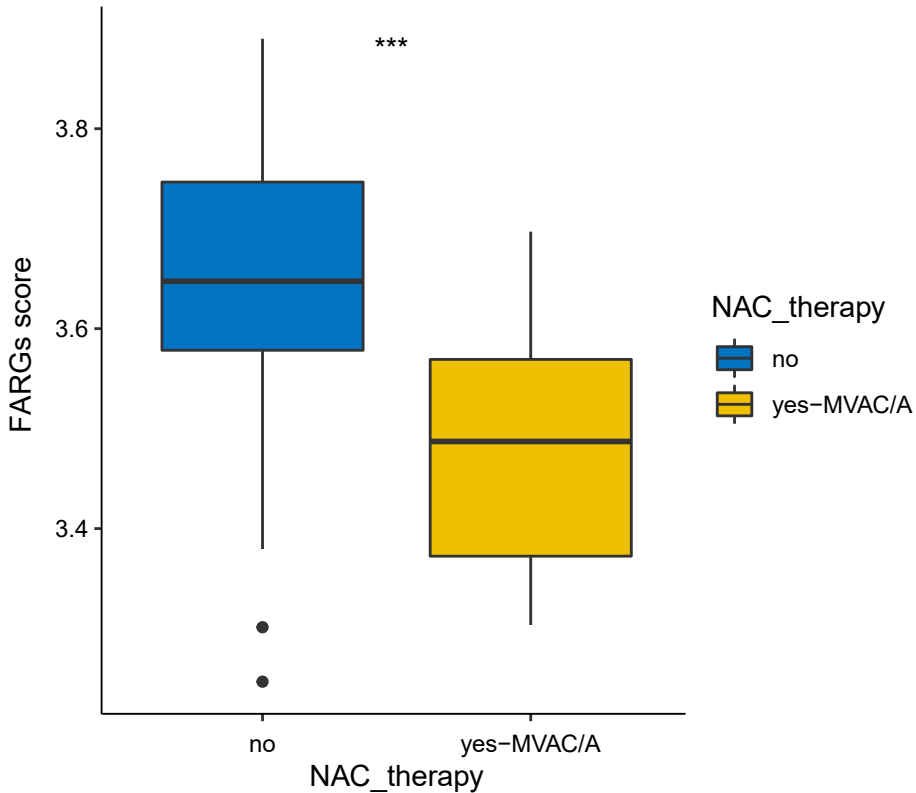

Supplement: Supplementary file 8 [file Image_8.pdf]
