# Supplementary material for: Comprehensive FGFR3 alteration-related transcriptomic characterization is involved in immune infiltration and correlated with prognosis and immunotherapy response of bladder cancer
Source: Front Immunol. 2022 Jul 26;13:931906. doi: 10.3389/fimmu.2022.931906 (PMC9360490; doi:10.3389/fimmu.2022.931906)
Supplement: Supplementary file 5 [file Image_5.pdf]

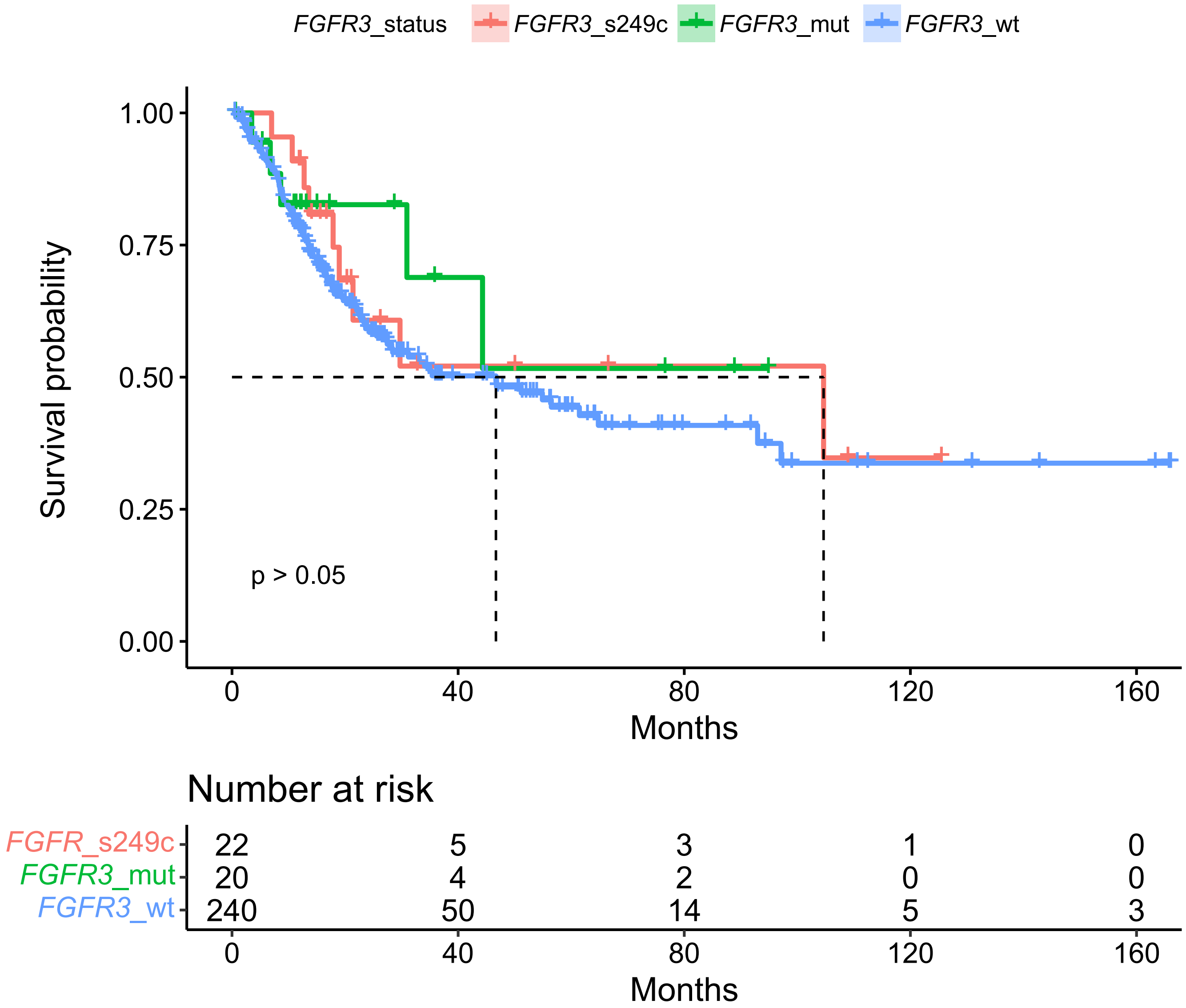
FGFR3_status
FGFR3_s249c
FGFR3_mut
FGFR3_wt
Survival probability
1.00
0.75
0.50
0.25
0.00
p > 0.05
0
40
80
120
160
Months
Number at risk
FGFR_s249c 22 5 3 1 0
FGFR3_mut 20 4 2 0 0
FGFR3_wt 240 50 14 5 3
0
40
80
120
160
Months
